# Spatial visual function in anomalous trichromats: Is less more?

**DOI:** 10.1371/journal.pone.0209662

**Published:** 2019-01-23

**Authors:** Ravid Doron, Anna Sterkin, Moshe Fried, Oren Yehezkel, Maria Lev, Michael Belkin, Mordechai Rosner, Arieh S. Solomon, Yossi Mandel, Uri Polat

**Affiliations:** 1 Department of Optometry and Vision Science, Hadassah Academic College, Jerusalem, Israel; 2 Goldschleger Eye Research Institute, Sackler Faculty of Medicine, Tel-Aviv University, Tel-Hashomer, Israel; 3 The School of Optometry and Vision Science, The Mina & Everard Goodman Faculty of Life Sciences, Bar Ilan University, Ramat-Gan, Israel; 4 Bar-Ilan Institute for Nanotechnology and Advanced Material (BINA), Bar Ilan University, Ramat-Gan, Israel; University of Sussex, UNITED KINGDOM

## Abstract

Color deficiency is a common inherited disorder affecting 8% of Caucasian males with anomalous trichromacy (AT); it is the most common type of inherited color vision deficiency. Anomalous trichromacy is caused by alteration of one of the three cone-opsins’ spectral sensitivity; it is usually considered to impose marked limitations for daily life as well as for choice of occupation. Nevertheless, we show here that anomalous trichromat subjects have superior basic visual functions such as visual acuity (VA), contrast sensitivity (CS), and stereo acuity, compared with participants with normal color vision. Both contrast sensitivity and stereo acuity performance were correlated with the severity of color deficiency. We further show that subjects with anomalous trichromacy exhibit a better ability to detect objects camouflaged in natural gray scale figures. The advantages of color-deficient subjects in spatial vision performance could explain the relatively high prevalence of color-vision polymorphism in humans.

## Introduction

Anomalous trichromat subjects have a different perception of colors and therefore are unable to distinguish between certain colors that can be resolved by normal color vision subjects. Color deficiency imposes significant limitations for daily life [1], such as selection of craft work [2], plant or flower recognition [2], or selection of ripe fruits and vegetables [2]. Importantly, 43% of dichromats and 29% of anomalous trichromats reported that their color deficiency affected their choice of occupation, and about one quarter of them reported having been precluded from an occupation, refused for recruitment to the police or armed forces, as well as being excluded from railways or companies specializing in electronics and telecommunication. Medical practitioners, including vision care providers, also reported some difficulties in identifying eye redness or skin rash [3–5]. Furthermore, since color is extensively used in various learning activities, color deficiency can be associated with difficulties in studying and has some psychological implications [6].

On the other hand, in contrast to the disadvantage of having color deficiency, previously described, during World War II and later, it was suggested that color-deficient observers could often penetrate camouflage that effectively deceives normal observers [7, 8]. Later studies [9–11] also reported selective advantages for individuals having color vision deficiency in various visual tasks, such as superior detection of color camouflaged textures [9] or better scotopic [11] or mesopic [10] vision (the latter was not found in other studies [12]). Similarly, dichromats display better performance in tasks involving high temporal frequency stimuli [13] and have better contrast sensitivity than do controls under both static and dynamic conditions [14]. Interestingly, when dichromat subjects were genotyped, visual acuity was enhanced in multi-gene dichromats [15] but not in single-gene dichromats. Nevertheless, not much information has been published on anomalous trichromats’ visual functions. We were therefore motivated to conduct a comprehensive study of basic visual functions in anomalous trichromats, the most common color deficiency, compared with normal subjects. First, we measured visual acuity, the most widely used clinical tool for measuring visual functions [16]. Second, we measured contrast sensitivity, which is recognized as a highly sensitive complementary tool for assessing visual functions and visual deficits [17–19] and represents the combined response of neurons in the primary visual cortex [20]. We further evaluated stereo acuity, which is defined as the ability to perceive depth due to binocular disparity,[21]. We speculated that, similarly to the dichromats’ superior performance, anomalous trichromat subjects would have superior visual functions over normal subjects.

## Methods

### Subjects

Anomalous trichromat subjects were recruited to the research by an advertisement stating that subjects with color deficiency are required for an experiment. Control subjects were similarly recruited to the research and were age- and education-matched to the anomalous trichromat subjects. All subjects were naïve to the specific research aims and were informed that the research was generally linked to vision. In order to avoid group bias, all subjects were tested using exactly the same protocol and methods, and by the same single experimenter (R.D), at the same time of the day throughout the entire duration of the research.

The Human Research Committee at the Sheba Medical Center approved the study. Informed written consent was obtained from all subjects. All experimental protocols were performed in accordance with the guidelines provided by the committee approving the experiments.

Subjects were included in the research if their corrected-to-normal visual acuity was 20/25 or better, which was tested by a modified Bailey–Lovie (LogMAR) chart (ETDRS) by a qualified optometrist.

### Color vision assessment

Qualitative diagnosis of color anomalous deficiency was assessed in two steps [22, 23]: We first used the Ishihara test, which is frequently used as a screening test to identify subjects with anomalous trichromacy [24]. Further quantification of the color vision anomaly was tested using the AO-HRR color test [23, 25], which can differentiate between protan and deutan, and can estimate the color deficiency severity. This test is of high validity in grading "mild" deficiency cases [26]; however, the grading of "medium" or "strong" cases can be inaccurate [22]. These tests are often used in clinical settings for screening in order to preclude subjects with color deficiencies from specific occupations that require normal color vision (this varies between countries) [25, 27].

### Apparatus and visual function testing

All visual stimuli were displayed as gray-level modulations on a Philips 107P color monitor. The experiments were controlled by a Dell PC with a NVIDIA Quadro FX 1400 graphic card. Screen resolution was 1024 x 768 pixels. The mean display luminance was 30 cd/m^2^ in an otherwise dark environment. Gamma correction was performed by measuring the screen luminance at 10 points by using a LS-100 Luminance Meter (Konica-Minolta) and correcting luminance to a linear fit. The procedure was repeated until the best fit was achieved. This approach did not involve bit loss. The stimuli were viewed from a distance of 300 cm for visual acuity, 150 cm for contrast sensitivity measurement, and 250 cm for the stereo acuity task.

Basic visual functions were tested by using customized computer software, which was previously reported by our group for determining visual acuity [28], contrast sensitivity [17], and stereo acuity [29, 30], which is briefly explored below.

Visual acuity was measured by a computerized test [31] with a tumbling-E pattern paradigm, during which subjects were asked to detect the "open" E direction (4 alternatives—up, down, right, and left) presented on a computer monitor. The test was performed under two conditions: 1) the target as a single E and 2) the target embedded in a matrix of tree rows (as in an ETDRS chart) of E letters spaced by one letter size, where subjects had to respond to the central E. Visual acuity was measured using an adaptive procedure, in which the pattern size and spacing were modified in 0.1 log unit steps; this was used to determine the minimal size needed for a 50% accuracy level (the chance level was 25%). Subjects were informed of a wrong answer by an auditory feedback after each trial throughout the experiment.

**Contrast sensitivity** (CS) stimuli were presented as gray-level images with vertical grating Gabor patches (GP). The spread of the Gaussian envelope was equated with the wavelength of the carrier^40^ to allow at least 2 cycles. Stimuli were measured under photopic conditions modulated from a background luminance of 30 cd/m^2^ with a spatial frequency of 6 and 9 cycles per degree (cpd) and under mesopic conditions (background luminance 0.1 cd/m^2^) with spatial frequencies of 3 and 6 cpd [17]. A contrast detection paradigm was used for contrast sensitivity measurements. Four blank circles were presented for an unlimited duration (static). The subjects were asked to detect the Gabor patches (target) in a four-alternative forced choice task in one of the 4 spatial locations (up, down, right, and left). Auditory feedback was given after each wrong response. The target appeared randomly in 1 of the 4 locations. Contrast detection thresholds were measured using a staircase method estimating the stimulus detection threshold at the 79% accuracy level [19]. This procedure was repeated for each spatial frequency in a randomized order to avoid bias of results or the confounding effects of fatigue and adaptation time. Photopic testing was performed first, followed by a short break. The mesopic condition was performed after 1 minute of dark adaptation, which enables the beginning of testing under the foveal mesopic condition [19].

Stereo acuity was measured similarly to our previous report [30] using a PC and stereo goggles (“Crystal eyes 3” by Stereo Graphics), allowing the presentation of different stimuli to each eye and with the subjects unaware of the eyes targeted on each trial. The subjects’ task was to decide whether a vertical line (0.63 by 2.5 mm, subtending 0.09 by 0.36 degrees, with a luminance of 63 cd/m^2^, and a 1-second presentation time) was perceived as “outward” or "inwards" compared to a reference line of similar size and luminance that was fixed. The stereo acuity threshold was estimated by calculating the threshold (79% accuracy level) from the psychophysical curve, as was previously reported by our group [30].

To measure the ability to detect camouflage objects in natural images under controlled conditions, we developed a figure-ground discrimination task [30]. This method was based on 5 real-world scenes, in which multiple target objects, such as animals, cannons, soldiers, or aircraft were embedded in a natural way (as reported previously by Sterkin et al., 2017). On average, 10 target objects were embedded in each image and their location was registered. Both the targets and background scenes were gray-level (uncolored) with a mean target size of 3.8 cm by 2.1 cm, subtending 5.4 by 3 degrees, and an image size of 19.1 cm by 27.3 cm, subtending 26.9 by 37.7 degrees. The task was to detect the targets by pointing at their location using a computer mouse. The contrast level of the whole image was gradually increased, from 2% up to 8%, in steps of 2%. Subjects were informed about the type of the target but not about the number of targets within each trial. One minute was allowed for detecting targets for each contrast level. The percentage of correct responses (PC) for each contrast level was calculated as the rate of hits (H) and false alarms (FA) according to Eq (1). Sensitivity was then calculated as d’.

PC=(H+(1-FA))/2 (1)

### Data analysis

We used the two-tailed unpaired t-test for comparing conditions between the anomalous trichromat and the control subjects (unless otherwise stated); the effect size was measured according to Cohen's d. Pearson's correlation was used for studying the correlation between visual functions and the anomalous trichromat severity.

## Results

Fifty-seven volunteers were screened for the study; 5 were excluded because of corrected visual acuity lower than 20/25 and the other 4 subjects were excluded because they were diagnosed as dichromats. Overall, 24 anomalous trichromat and 24 normal color vision subjects, aged 18 to 33 years (anomalous trichromat mean age = 26 years, control mean age = 24 years) participated in the study; all participants were graduate students.

Based on the AO-HRR evaluation, 18 subjects were diagnosed as having a mild deuteranomalous defect and 6 subjects were diagnosed as having a mild protanomalous defect. S1 Fig illustrates the distribution of color deficiency severity among subjects. Higher scores indicate more severe color deficiency.

**Fig 1:**
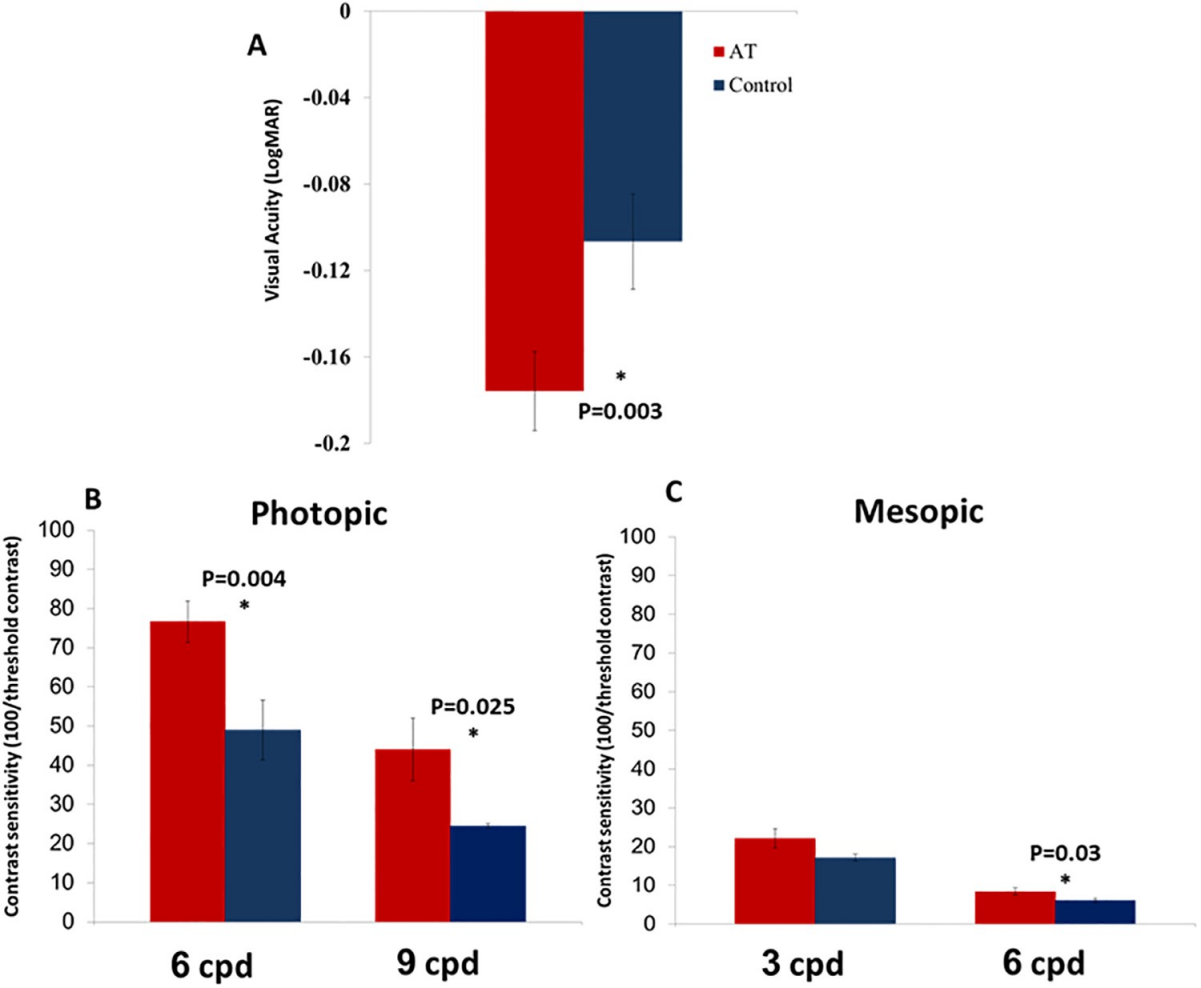
Visual acuity and contrast sensitivity in normal and anomalous trichromat subjects. ***(a)*** Visual acuity is represented in LogMAR in anomalous trichromat subjects (red) and in normal color vision controls (blue). Lower values represent higher visual acuity (logMAR = 0 is equal to 20/20). Error bars represent the standard error of the mean. ***(b)***. Photopic contrast sensitivity at 6 and 9 cpd (red for anomalous trichromats and blue for controls). ***(c)***. Mesopic contrast sensitivity at 3 and 6 cpd (red for anomalous trichromats and blue for controls). Error bars represent the standard error of the mean.

In support of our hypothesis, we found that subjects with Anomalous trichromacy performed better in all visual function tests, compared with subjects with normal color vision. The mean visual acuity was significantly better (t (46) = -3.11, p = 0.003, d = 0.13) in anomalous trichromat subjects compared with normal age-matched controls by 0.075 LogMAR units (Fig 1a). Importantly, this outperformance by anomalous trichromats was observed despite the ceiling effect that was expected due to the subjects’ young age and since both groups had an average visual acuity better than 0 logMAR (20/20). Note that an above-normal visual acuity level in healthy young adults was previously reported when visual acuity was measured with LogMar charts [32].

A similar superior performance of anomalous trichromat subjects was found for contrast sensitivity under photopic conditions at both 6 cpd (t (46) = 3.14, p = 0.004, d = 1.16) and 9 cpd (t (46) = 2.37, p = 0.025, d = 0.89) (Fig 1b) and under mesopic conditions at only 6 cpd (t (46) = 2.27, p = 0.03, d = 0.85). Contrast sensitivity at 3 cpd was higher in anomalous trichromats, but the difference was not statistically significant (t (46) = 1.88, p = 0.07, d = 0.7) (Fig 1c). Note that the control's contrast sensitivity performance in our experimental set-up was similar to previous reports of contrast sensitivity obtained by Gabor stimuli (e.g., [17]). Overall, our results show that anomalous trichromat individuals are more sensitive to subtle changes in gray levels, irrespective of the lighting conditions.

**Fig 2:**
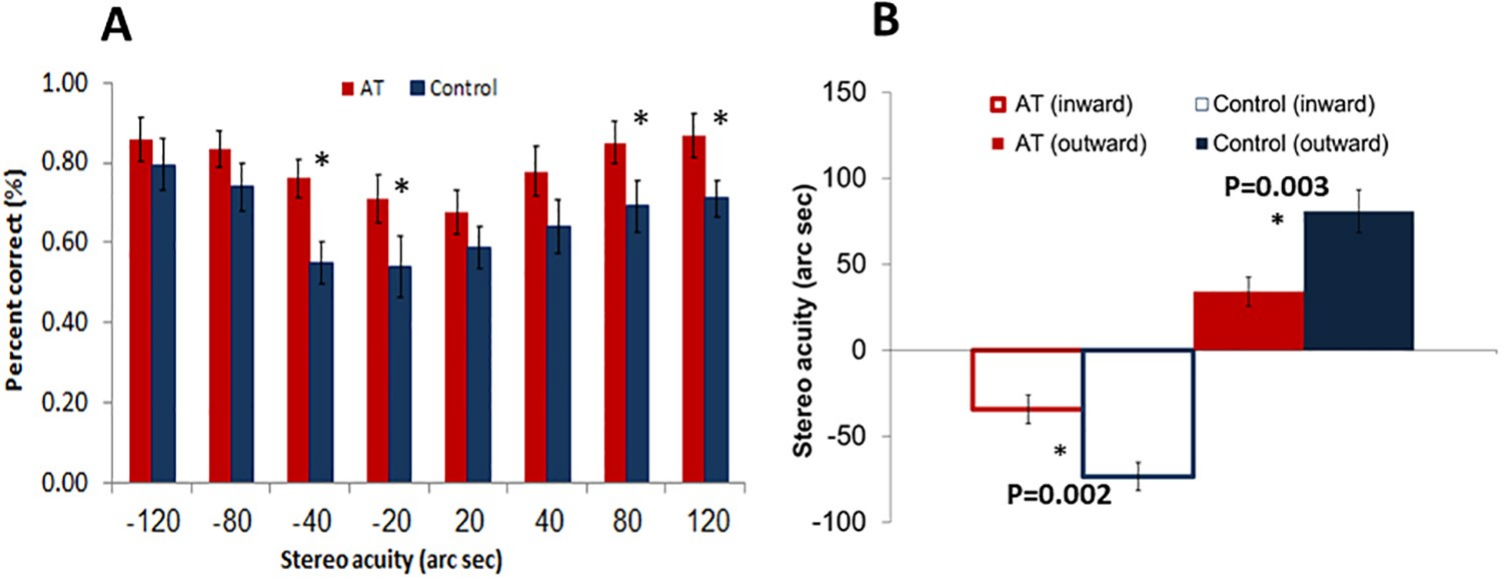
Stereo acuity performance: Stereo acuity in anomalous trichromat subjects and in normal color vision controls. ***(a)***. Percent correct vs. stereo acuity in anomalous trichromats (red) and controls (blue). ***(b)***. Stereo acuity threshold: "outward" (red—anomalous trichromats, blue—controls) and "inward" (vacant red—anomalous trichromats, vacant blue—controls). Error bars represent the standard error of the mean.

In addition to the superior performance of visual acuity and contrast sensitivity, anomalous trichromat subjects also performed significantly better in a stereo acuity test. Compared with normal subjects, anomalous trichromat subjects had, on average, higher rates of correct responses in all disparities (Fig 2a, t (46) = 3.24, p = 0.005, d = 1.71) and a significant different was shown for a near-threshold disparity of -20 (t (46) = 3.16, p = 0.002, d = 1.37) and -40 (t (46) = 1.82, p = 0.04, d = 1.75), which support the possibility of superior stereo processing in the AT. Nevertheless, statistically significant differences were also found for the larger disparities of +80 (t (46) = 2.01, p = 0.03, d = 1.78), and +120 (t (46) = 2.35, p = 0.01, d = 0.96), which could arise from monocular cues. Overall, a significantly better average stereo acuity threshold (Fig 2b) for both inward (t (46) = 3.47, p = 0.002, d = 1.41) and outward (t (46) = -3.24, p = 0.004, d = 1.3) conditions was found.

**Fig 3:**
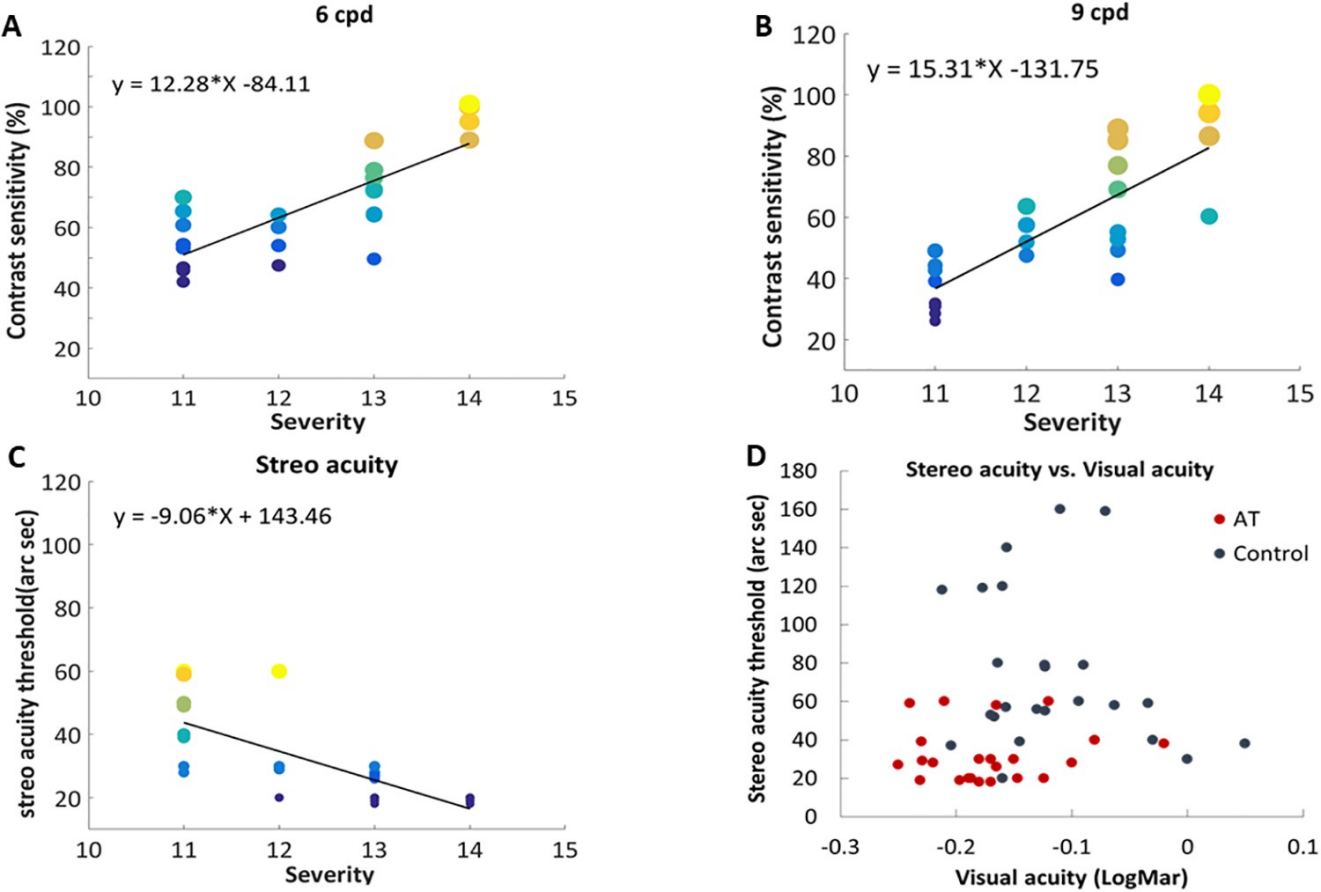
Correlation of visual performances. ***(a)***. Correlation between contrast sensitivity (%) at 6 cpd and the severity rate (the last place where the subject made an error) of the red-green deficiency. Line, linear regression. The dot’s diameter is scaled by frequency and dots are further colored for clarity ***(b)***. Correlation between contrast sensitivity (%) at 9 cpd and the severity rate (the last place where the subject made an error) of the red-green deficiency. Line, linear regression. The dot’s diameter is scaled by frequency and dots are colored for clarity. ***(c)***. Correlation between the stereo acuity threshold (arc sec) and the severity rate (the last place where the subject made an error) in the red-green deficiency. Line, linear regression. The dot’s diameter is scaled by frequency and dots are colored for clarity. ***(d)***. Correlation between visual acuity and the stereo acuity threshold (arc sec) in all anomalous trichromat and control subjects.

In order to determine whether the superior spatial functions of anomalous trichromat subjects are related to the severity of the color deficiency, we calculated the correlation between the subjects’ spatial functions and the color degree of severity, i.e., severity grade (the last place where the patient made an error using the HRR test). Statistically significant correlations (Fig 3a and 3b) were found between contrast sensitivity and the severity of color deficiency for both spatial frequencies at 6 cpd (r(22) = 0.790, p<0.001) and 9 cpd (r(22) = 0.760, p<0.001). Similarly, a significant correlation was also found between the subjects’ stereo acuity thresholds and the color deficiency severity grade (Fig 3c, r(22) = -0.725, p<0.001). In contrast, a weak correlation, but not statistically significant, was found between visual acuity and the color severity grade (r(22) = -0.34, p = 0.1); data not shown.

We further investigated whether the higher stereo acuity observed in anomalous trichromats is independent of the higher visual acuity observed in this group. Fig 3d depicts a scatter plot with a distribution of stereo acuity at the expected range [33]. It can be seen that stereo acuity was better in anomalous trichromats, compared with normal control subjects with similar visual acuity (Fig 3d). Thus, apparently the superior stereo acuity of anomalous trichromat subjects is not caused by their better visual acuity, but instead, is an additional independent advantage of being an anomalous trichromat. Indeed, no correlation was found between visual acuity and stereo acuity for both anomalous trichromats and normal subjects (r(22) = -0.23, p = 0.28 and r(22) = 0.05, p = 0.81, respectively).

Taken together, the results support our hypothesis that basic spatial visual functions (visual acuity, contrast sensitivity, and stereo acuity) of anomalous trichromat individuals are superior to those of normal color vision controls.

**Fig 4:**
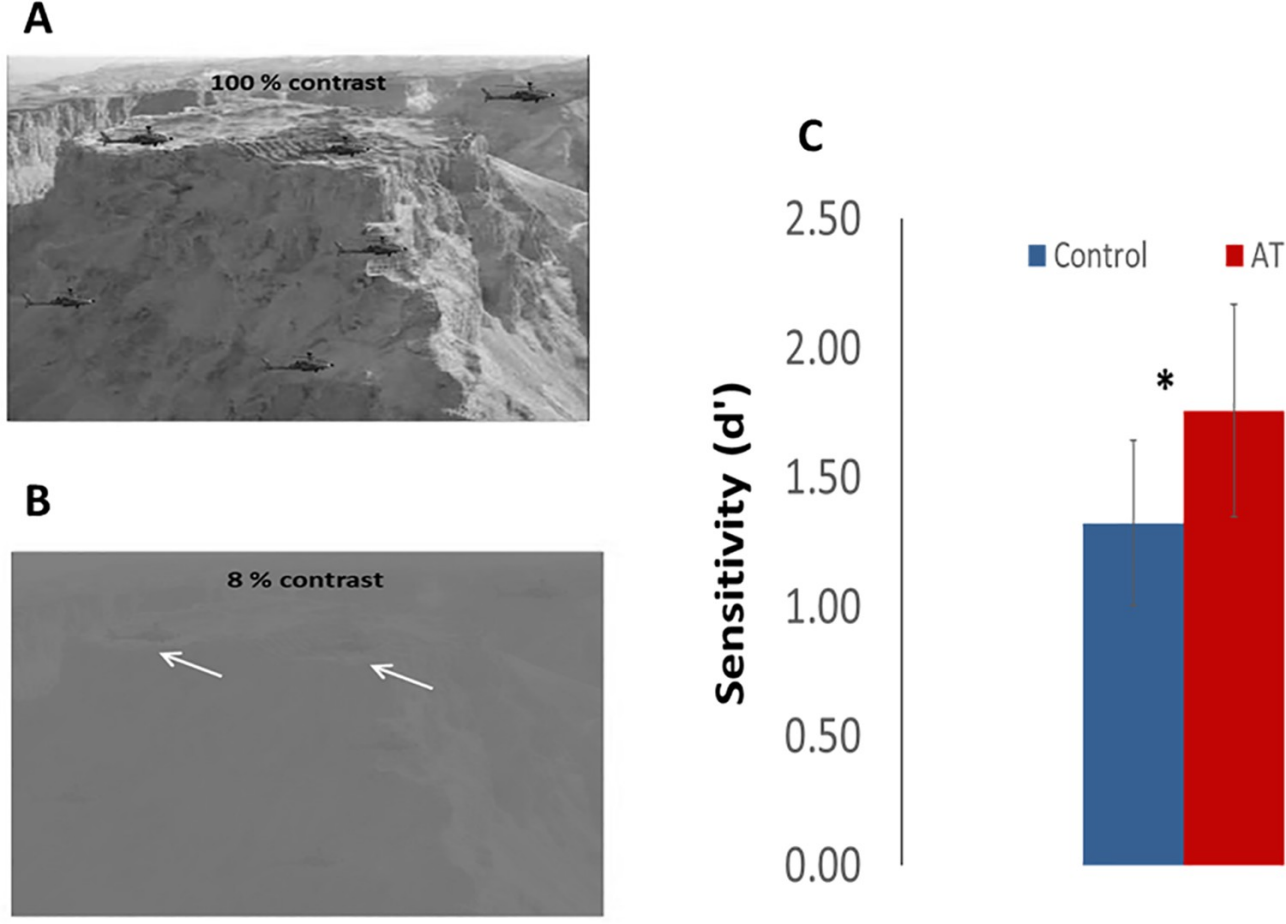
Detecting camouflage. ***(a)***. An example of one aerial image used by our custom software at a contrast of 100% (not used in the test) and the same image ***(b)*** with 8% contrast (the highest contrast used for testing). ***(c)***. Mean sensitivity (d’) for the 5 images. Sensitivity was calculated per subject per image and then averaged for each group. *Aerial view of Masada (Andrew Shiva/Wikipedia/CC BY-SA 4*.*0)*.

To further investigate whether the superior performance in basic visual functions found in anomalous trichromats is also associated with better performance in complex spatial tasks, we developed custom software that measures the ability to detect camouflaged objects in actual aerial photography images under controlled conditions (see Methods)[30]. To this end, five images with increasing contrasts (from 2 to 8%) were tested on 9 anomalous trichromat and 9 control subjects. Fig 4 presents an example of an image at 100% (Fig 4a) and 8% contrast (Fig 4b). Fig 4c depicts the average sensitivity for 4 to 8% contrast (d’) that was significantly higher for anomalous trichromats (1.75 ± 0.41) compared with control subjects (1.32 ± 0.31) (t (16) = -2.49, p = 0.02, d = 1.39). Two-way repeated measures ANOVA was conducted on the influence of groups (AT and control) and contrast (four levels) on sensitivity to identify camouflage (d’). The main effect for the groups yielded an F ratio of F(1, 44) = 7.03, p<0.02, indicating a significant difference between AT and control. The main effect for contrast yielded an F ratio of F(1, 44) = 62.21, p<0.001, indicating a significant difference between contrast levels. The interaction effect was not significant, F(1, 44) = 1.34, p = 0.26. The results indicate that anomalous trichromat individuals can detect low-contrast objects, which could not be seen by individuals with normal color vision, consistent with previously reported superior camouflage detection in dichromats [9, 34].

## Discussion

This study shows the superior performances of anomalous trichromat individuals over normal color-vision subjects in basic spatial visual tasks: visual acuity, spatial contrast sensitivity, and stereo acuity. The observed robust performances of these basic visual functions in anomalous trichromats can be explained by visual processing modifications at different levels. One possible explanation is the decrease in chromatic noise, which is normally caused by both longitudinal [14] and transverse [35] chromatic aberrations at the levels of eye optics and photoreceptors [15], as is suggested by several authors [14, 15]. Importantly, it was reported [15] that increased visual acuity was found only in multi-gene dichromats and not in single-gene dichromats. The authors suggested that the expected increase in visual acuity caused by the absence of chromatic aberration in both single-gene and multi-gene dichromat genotypes is contrasted by plausible abnormal retinal wiring development occurring in single-gene but not in multi-gene dichromats. Note that in anomalous trichromats, the photoreceptor density and wiring are expected to be normal. Indeed, in line with this, in our anomalous trichromat subjects, the decrease in achromatic noise in the presence of otherwise normal retinal wiring is probably a major cause of the increased visual acuity and contrast sensitivity in our anomalous trichromat subjects.

Another possible mechanism for the increased spatial visual functions observed in anomalous trichromats is reduced red-green opponency, as was suggested [36, 37] and also supported by the findings of Sharpe, who reported an increase in luminance contrast [13] to high (but not for low) temporal frequency-modulated targets.

It was also suggested that deuteranomalous subjects possess post-receptorial amplification when processing chromatic information [38]. We suggest that post-receptorial amplification, which is associated with reduced color information in anomalous trichromats, also affects the achromatic processing in this group. It was further suggested by Janaky [14] that the enhanced luminance signal in dichromats can result in plastic changes in the visual cortex, such as an increased number of cortical neurons devoted to luminance processing, which could contribute to the increased contrast sensitivity in the high acuity channels. It is also possible that neurons in posterior area V4, normally dedicated to color processing [39], can now be engaged in the processing of achromatic information. It should be mentioned that both Sharpe and Janaky reported results for dichromats rather than for anomalous trichromats. Nevertheless, we suggest that similar plasticity changes and processing may occur in anomalous trichromats.

The severity of color deficiency in our subjects was correlated with both contrast sensitivity and stereo acuity, but not with visual acuity (Fig 3a–3c). Our data further show that anomalous trichromat subjects had increased stereo acuity, compared with normal subjects regardless of their increased visual acuity and contrast sensitivity (Fig 3d), thus suggesting that cortical processing on the spectrally shifted retinal input could contribute to the enhanced performance. The outperformance of stereo acuity could arise from the greater contrast sensitivity observed in the anomalous trichromat group, which was suggested in several studies [40, 41]

In addition to the superior performance of anomalous trichromats in basic vision tasks, they also outperformed normal subjects in the more complex task of detecting camouflaged targets. Importantly, better color camouflage breaking in color-deficient (dichromat) subjects was reported by Troscianco [42] and Morgan [9], who suggested that dichromats are less susceptible to color interferences when performing segregation tasks. Similarly, better detection of color camouflage targets or insect capturing was reported for dichromats or anomalous trichromat non-human primates [34, 43, 44]. Interestingly, it was found that dichromats’ visual memory was similar to that of normal color subjects even when chromatic pictures were presented as memory targets [45]. Our results indicate that even under conditions where color information is not available (our images were achromatic), color-deficient subjects found significantly more hidden targets compared with normal subjects, probably as a result of their better segmentation capabilities.

Though superior performance in breaking achromatic camouflage was found to be associated with superior stereo function [46], the camouflage test in our study did not utilize a 3D task. We therefore suggest that the superior camouflage break in the AT could arise from better monocular spatial vision performance observed in this group, which may provide better stereo vision.

Another suggestion, inspired by computer vision, is that detection of the gray-level objects may be employed by the visual system to break the camouflage [47]; hence, the enhanced contrast sensitivity in anomalous trichromats may directly enhance the task of breaking the camouflage.

Importantly, in the current research, we used two clinically available and acceptable methods for color vision classification [23, 25], but not the Rayleigh match or genetic analysis [23, 25, 48], which are considered the gold standard methods for research purposes. Note that the main aim of this research was to study the visual performance of subjects who are classified as anomalous trichromats in clinical settings by using a test widely used in clinical practice and during occupational screening (e.g.,[49]). If we used genetics or the Rayleigh match, we would probably have found that our psychophysical tests misclassified a few normal subjects as having anomalous trichromacy. Thus, our results, in the worst case, present an under estimation of the visual abilities of the anomalous trichromat subjects. Nevertheless, we suggest that future research studying the effect of color vision on spatial and stereo visual performance should use Rayleigh match and genetic profiles of the subjects.

Another potential possible bias is over-motivation, which is an important factor that may affect the results of psychophysical tests [50]. Though we cannot completely rule out that our anomalous trichromat subjects were over-motivated by their deficiency, the study was designed to minimize over-motivation pressure, since the subjects were naïve to the specific research aims and the procedures were identical between the two groups. In addition, the significant correlation between the severity of color deficiency and the contrast sensitivity at 6cpd, 9cpd, as well as visual acuity, suggest a physiological rather than a motivational effect. Finally, both groups were further motivated to achieve high performance during the current study, in order to be suitable for future studies in the laboratory.

Interestingly, in line with the high prevalence of color deficiency and the above-mentioned reported superior function in color-deficient subjects and in non-human primates, it was suggested [9, 11, 51] that color vision polymorphism could have been maintained during evolution because of the potential advantages of cooperation between normal-color and color-deficient individuals when foraging in groups. It could be beneficial for the cooperative activity to have subjects with good color vision, along with color-deficient individuals, who can penetrate color or achromatic camouflage, or who function better under low illumination and contrast conditions. Based on our results, it could therefore also be possible that the superior basic visual function and camouflage-breaking capabilities could benefit anomalous trichromat subjects when directed to specific occupations requiring good spatial and stereo acuity, mainly where visual tasks are performed in a collaborative effort with normal color vision subjects.

## Supporting information

S1 Fig AO-HRR pseudoisochromatic plate segmentation. Subjects were diagnosed according to their AO-HRR plates. Twenty-four subjects were diagnosed as anomalous trichromats (18 deuteranomaly and 6 protanomaly subjects).
(TIF)

S1 Data Row data—an excel file. (XLSX)
