# Supplementary figures and images for: Spatial visual function in anomalous trichromats: Is less more?
Source: PLoS One. 2019 Jan 23;14(1):e0209662. doi: 10.1371/journal.pone.0209662 (PMC6343896; doi:10.1371/journal.pone.0209662)

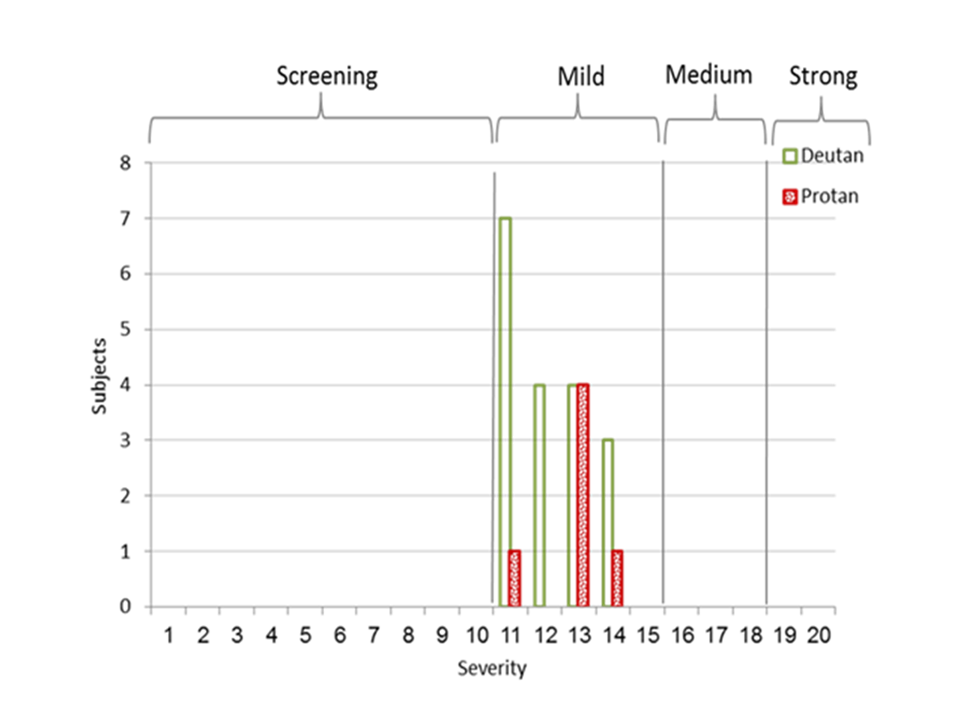

Supplement: S1 Fig — Subjects were diagnosed according to their AO-HRR plates. Twenty-four subjects were diagnosed as anomalous trichromats (18 deuteranomaly and 6 protanomaly subjects). (TIF) [file pone.0209662.s001.tif]
